# Fighting COVID-19 in the West Africa after experiencing the Ebola epidemic

**DOI:** 10.34172/hpp.2021.02

**Published:** 2021-02-07

**Authors:** Pourya Gholizadeh, Moussa Sanogo, Amadou Oumarou, Maad Nasser Mohamed, Yacouba Cissoko, Mamadou Saliou Sow, Pasquale Pagliano, Patassi Akouda, Sid’Ahmed Soufiane, Akory Ag Iknane, Mamadou Oury, Safiatou Diallo, Şükran Köse, Sounkalo Dao, Hossein Samadi Kafil

**Affiliations:** ^1^Research Center for Pharmaceutical Nanotechnology, Tabriz University of Medical Sciences, Tabriz, Iran; ^2^Drug Applied Research Center, Tabriz University of Medical Sciences, Tabriz, Iran; ^3^Faculté de Médecine, de Pharmacie et d’Odonto-Stomatologie (FMPOS), University of Bamako, Bamako, Mali; ^4^Faculte des sciences la santé de l universite Dan Dicko DanKoulodo de Maradi, Niger; ^5^Service des maladies infectieuses et tropicales de l’hôpital général peltier, Djibouti; ^6^Faculty of Medicine and Dentistry, University of Sciences, Techniques and Technologies of Bamako (USTTB), Bamako, Mali; ^7^Service des Maladies Infectieuses, Hôpital National Donka, CHU Conakry, Centre de Recherche et de Formation en Infectiologie de Guinée (CERFIG), Guinea; ^8^Departement of Medicine, University of Salerno, Salerno, Italy; ^9^CHU Sylvanus Olympio, Universitie delome, Togo; ^10^Faculte de Medecine de Nouakchott, Muritanie; ^11^Institut National de Santé Publique, Bamako, Mali; ^12^Department of Infectious Diseases and Clinical Microbiology, University of Health Sciences, Tepecik Training and Research Hospital, İzmir, Turkey

**Keywords:** Africa, COVID-19, SARSCoV-2, Epidemiology, Ebola virus, diagnosis, pandemic

## Abstract

Coronavirus disease 2019 (COVID-19) dissemination occurred from December 2019 and quickly spread to all countries. Infected patients with COVID-19 have had a wide range of symptoms, ranging from mild to severe illness. The most mortality was observed in patients with underlying disease and over 45 years. World statistics have shown that the COVID-19 outbreak is most expanded in Middle Eastern, West Asian, European, North, and South American countries, and is least expanded in African countries. Therefore, the aim of the paper was the evaluation of six African countries including Mali, Mauritania, Niger, Guinea, Togo, and Djibouti to find why this disease is least expanded in African countries. Study was conducted by Questioner for countries health organizers to define their different aspect exposure and fight with COVID-19 including epidemiology, clinical aspects of the disease, case definitions, diagnosis laboratory confirmation, and referral of cases by the portal of entry, case management, and disease prevention in these countries. According to this opinion review, due to the low international flights and low domestic travel, the spread, and prevalence of COVID-19 was low and the return of the immigrants of these countries has caused the spread of COVID-19 among these countries. Experience, preparation, and impact of previous infections epidemic such as the Ebola virus epidemic would have beneficial, which have promoted certain reflexes among people that cause low dissemination in these countries.

## Introduction

Severe acute respiratory syndrome corona virus-2 (SARS-CoV-2) was emerged from Wuhan, China in December 2019 and was spread worldwide as a one of the biggest pandemics in the human history.^1^ Coronaviruses are enveloped positive-sense and non-segmented RNA viruses, which belong to the family Coronaviridae. These viruses are broadly distributed among humans and mammals.^2,3^ Middle-East respiratory syndrome corona virus (MERS-CoV) and severe acute respiratory syndrome corona virus (SARS-CoV-1) were the most recent of epidemics caused by coronavirus members.^4,5^ They had 37% and 10% mortality rates, respectively. Both MERS-CoV and SARS-CoV-1 were originated from animals and were directly transmitted to humans from dromedary camels and market civets, respectively.^6-8^ The SARS-CoV-1 outbreak involved more than eight thousand patients and spread to 29 countries globally during 2002-2003,^6,7^ while MERS-CoV has emerged in Middle Eastern countries during 2012.^8^ However, SARS-CoV-2 quickly spread to all countries. On January 30, 2020, the World Health Organization (WHO) declared the COVID-19 coronavirus epidemic is a public health emergency of international concern.^9^ The symptoms of the disease vary from mild symptoms to severe illness, which may appear 2-14 days after exposure to the virus including fever, fatigue, dry cough, sore throat, loss of taste or smell, chills, repeating shaking with chills, headache, muscle pain, and in severe illness dyspnea, shortness of breathing or difficulty breathing.^10-14^ According to the data, all ages can be infected with COVID-19 but most of the infections and mortality of infected patients were observed in patients over 60 years old and patients with underlying diseases such as lung disease, cancer, diabetes, immunodeficiency, hypertension, and heart diseases, kidney, liver and gastrointestinal diseases, which are the most high risk individuals against COVID-19. The national guidelines of the countries for the management of COVID-19 derived from their importance in the major public health problem posed by this disease. It represents a threat to health security, with a significant socio-economic impact. The guidelines are adapted from the experiences of heavily affected countries, and several aspects of infection with the new coronavirus including epidemiology, clinical aspects of the disease, case definitions, diagnosis laboratory confirmation, referral of cases by the portal of entry, case management, and disease prevention.^15^ World statistics have shown that the COVID-19 outbreak is most expanded in Middle Eastern, West Asian, European, North and South American countries, while is least expanded in African countries. Therefore, the aim of the paper was the evaluation of five African countries including Mali, Mauritania, Niger, Guinea, Togo to find why this disease is least expanded in African countries. Study was conducted by Questioner for countries health organizers to define their different aspect exposure and fight with covid-19 including epidemiology, clinical aspects of the disease, case definitions, diagnosis laboratory confirmation, and referral of cases by the portal of entry, case management, and disease prevention in these countries.

## Geographical situation and demographic factors

Mali, a landlocked country in West Africa, covering an area of 1 266 491 km^2^, which is located in the pivotal zone between the Sahel and the Sahara. It shares thousands of kilometers of borders with 8 neighboring countries: Algeria in the North; Niger and Burkina Faso in the east; Ivory Coast and Guinea to the south; Senegal and Mauritania to the west. Its population in 2018 was estimated at 19 737 858.

Mauritania is another neighboring country of Mali and covering an area of more than 1 000 000 km^2^, with a population of 4 403 313 in 2018.

Niger, one of the largest countries in West Africa, bordered by 7 countries with which it shares borders with thousands of kilometers. It has an area of 1 266 491 km^2^, with a population of nearly 23 million.

The Republic of Guinea, a country in West Africa, covering an area of 245 857 km^2^, and shares hundreds of kilometers of borders with each of these six bordering countries: Senegal in the North, Guinea -Bissau to the North-West, the Atlantic Ocean to the West, Sierra Leone and Liberia to the South, the Ivory Coast to the East and Mali to the North. Its population in 2018 is estimated at 14 216 998. This highly mobile population has maintained very solid trade relations with, among others, the Chinese people since the establishment of diplomatic relations on October 4, 1959, with countries in the sub-region and elsewhere. These population movements increase the risk of the spread of epidemics in Guinea.

Togolese Republic (Togo) is a country in West Africa bordered by Burkina Faso to the north, Benin to the east, and Ghana to the west. This country covers 57 000 km^2^, with a population of approximately 7.9 million. It is a sub-Saharan and tropical nation with a climate that provides good growing seasons, therefore, its economy depends highly on agriculture.

Niger, by virtue of its geographical position and its immensity, knows significant mobility of the West African population with regard to the exchanges it maintains with the seven border countries all in the epidemic, the significant flows of migrants to European countries and the rest of the world, the risk of the epidemic becoming widespread is real. The population of these countries is very mobile and due to the geographical situation of the countries, is exposed to epidemics.

These countries have migratory culture, several hundred thousand Malians, Mauritanians and Nigeriens live outside the country. In recent years, these countries have also experienced displacements of the refugee population fleeing insecurity, an influx of foreigners within the framework of peacekeeping operations, as well as, a significant flow of migrants from sub-Saharan Africa on route to European countries. These population movements increase the risk of the spread of epidemics.

## History of COVID-19 in the countries and strategic plan to fight

Mali after almost all of its neighboring countries finally notified its first case of COVID-19 on March 25, 2020. Before this date, a strategic plan to fight against COVID-19 was drawn up in order to contain this major public health problem, which represents a threat to health security, with a significant socio-economic impact. The Malian guidelines were adapted from the experiences of heavily affected countries and several aspects of infection with the new coronavirus (SARS-CoV-2) including epidemiology, clinical aspects of the disease, case definitions, laboratory diagnosis confirmation, and referral of cases by the portal of entry, case management and disease prevention. These guidelines indicate to health workers, the guidelines for appropriate and coordinated case management in the health context of Mali. From the onset of the outbreak caused by SARS-CoV-2 in China and long before the WHO declared the pandemic, Mali was on alert. The first measures were checks at airports and informing the populations. On March 5, 2020, a national plan to fight COVID-19 is available. On March 19, the Superior Council of National Defense closed airports, schools, banned gatherings of more than 50 people. On March 24, 2020, two suspected cases of COVID-19 were notified 1 by the region of Koulikoro and 1 by that of Kayes. Their laboratory confirmation led authorities to declare the COVID-19 epidemic in Mali on March 25, 2020. Subsequently, the epidemic spread to other parts of the country. On April 05, 2020, the report from the Ministry of Health showed 45 confirmed cases (including 16 imported) and 5 deaths.^16^

COVID-19 arrived in Lomé (Togo) on March 2, 2020, from Europe. The women started presenting some clinical symptoms on February 25. The clinical condition was judged to be mild. She was put in isolation in hospital on March 6, 2020, in the first COVID-19 center. She received antimalarial treatment and vitamin C during her hospitalization; she was discharged from the hospital with two real-time polymerase chain reaction (RT-PCR) tests negative 48 hours apart for SARS-CoV-2. Contacts were traced and kept under surveillance among those at home. There were 14 in total. At this time, the National Institute (INH) was the only laboratory in Togo where the confirmation by PCR tests was carried out. In addition to contact tracing in Togo, on the official notification of this first in Togo, non-pharmaceutical measures were announced at the highest level of the state. Nonpharmaceutical interventions were grouped into 4 major categories: declaration of the state of emergency, school closure, cancellation of public gatherings; closure of places of religious worship, containment of the population and isolation and quarantine, and curfew from 8 p.m. to 5 a.m. Clinically, after the announcement of laboratory results, the patient was isolated, and the course of the patient traced with contact tracing.

The number of alert cases 3921 in October 2020 and 3880 cases were investigated.^16^ In this group 2120 were confirmed. 1561 cases were cured, a proportion of 73.63% among confirmed cases. The number of deaths was 51 (2.41%). Nowadays the numbers of active cases are 508. Twenty- one women were pregnant. One hundred and thirty-two confirmed cases (including 11 actives) are staff in the health sector: 116 health professionals (44 nurses, 30 doctors, 20 health technicians, 7 midwives, 6 nursing assistants, 2 pharmacists) and 16 staff support. The number of tests carried out since March 2020 is 109,960, i.e. 142 tests per 10 000 inhabitants.

On February 21, 2020, the first case of suspected coronavirus in Mauritania was evoked in a Chinese patient in a private clinic. At the time the diagnosis was not possible in Mauritania, the sample was sent to the Pasteur Institute in Dakar in Senegal, where the H1N1 was isolated. Immediately after there was an implementation of response measures by the Ministry of Health: the creation of an interministerial crisis unit, establishment and equipment of a COVID-19 care center and its attachment to the national public health emergency operations center, strengthening of the diagnostic capacities of the INRSP (Institut National de Recherche en Santé Publique) Virology laboratory, for carrying out diagnostic tests for COVID-19 on-site with a deadline for delivery of results in less than 24 hours. Mauritania has continued to strengthen its response capacities to face the pandemic and thus prevent the spread of the virus on the national territory: the establishment of teams to take charge of travelers coming from virus circulation areas and the identification of containment sites, establishment of operational teams to monitor confined people.

The Ministry of Health and Public Hygiene of Guinea, through the National Health Security Agency (ANSS), has set up a system for managing air, sea, and land transport flows, monitoring, communication on means of preventing COVID-19. The health authorities have also made available a national repository on the overall management of COVID-19 and, with the support of partners, trained health care providers from the various epidemiological treatment centers already in existence since the onset of the disease Ebola virus across all regions of the country.

As soon as the pandemic was declared by China, President Djibouti initiated the creation of a committee to fight against COVID-19. Before the first confirmed case, a national management guide was established. The Djiboutian population has been sensitized; barrier gestures are initiated and ordered by the President of the Republic. On March 17, the country was almost closed and the sky cordoned off. Containment by neighborhoods was quickly implemented in the capital, where 80% of Djiboutians live. Then on March 18, the first case (Spanish military) was confirmed and then quickly evacuated. The epidemics set in continuously from March 22, two Djiboutian returning from France, and Turkey tested positive.

## The first case of COVID-19 in the countries

The first case in Mali is a female subject, aged 49, Malian commuting between France and Mali. She arrived in Bamako on March 12, 2020, from Paris. She started presenting an infectious picture on March 17 and respiratory on March 22, which led to the diagnosis of COVID-19 on March 24, 2020. She had a history of hypertension and was overweight. She was put in isolation in hospital on March 25, 2020, in the first COVID-19 center. She received treatment with chloroquine phosphate and azithromycin according to the national protocol, she had been treated for associated malaria during her hospitalization and on April 14, 2020, she has discharged from hospital with two RT-PCR tests negative 24 hours apart for SARS-CoV-2. On March 13, 2020, Mauritania recorded its first case of COVID-19 in an expatriate worker of a mining company who came from Australia and transited through Europe. The first cases detected in Niger go back to March. Niger, like other countries, has developed a national response plan against the spread of COVID-19 and a document of National Case Management Guidelines. Niger, through its national response plan, envisaged a multisectoral response strategy. Nigeriens do not know the exact date of re-entry of the first case. They are in the provinces with 600 km far from the place of diagnosis. The suspects (3) are male according to the committee set up by the Ministry of Public Health. These are Nigeriens who have stayed in France.

**Figure 1:**
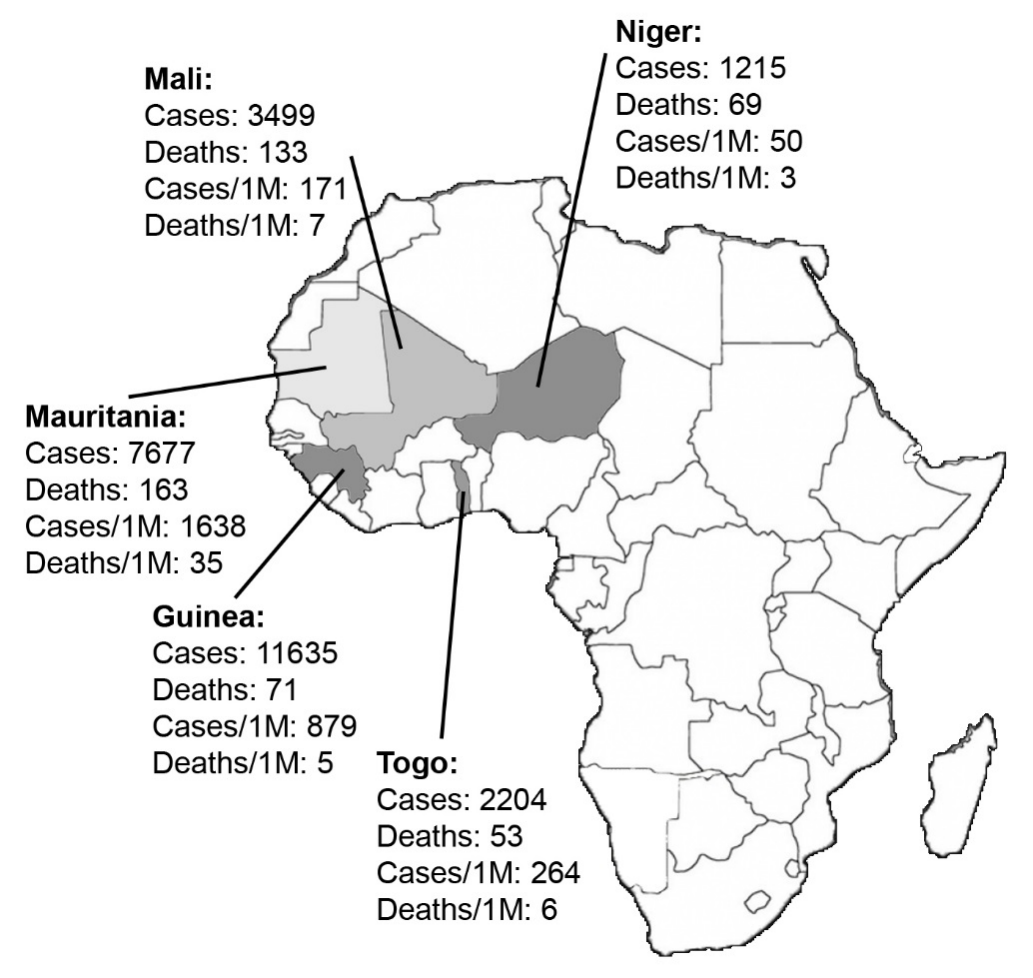
Reported cases and deaths in Mali, Mauritania, Niger, Guinea, and Togo until 27 October 2020.^16^

The first case in Guinea is a female subject, aged 49, Belgian, employee of the European Union delegation in Guinea, from France. She arrived in Conakry on March 03, 2020, from Paris. She started presenting a respiratory picture on March 10 and then confirmed a diagnosis of COVID-19 on March 12, 2020. She has no particular pathological history. She was put in isolation in hospital on March 12, 2020, in the epidemiological center of Nongo. She received treatment with Chloroquine Phosphate and Azithromycin according to national protocol. The outcome was favorable with discharge from the hospital after two negative RT-PCR tests 24 hours apart for SARS-CoV-2 (Figure 1).

## Date of laboratory diagnosis in the countries

**Figure 2:**
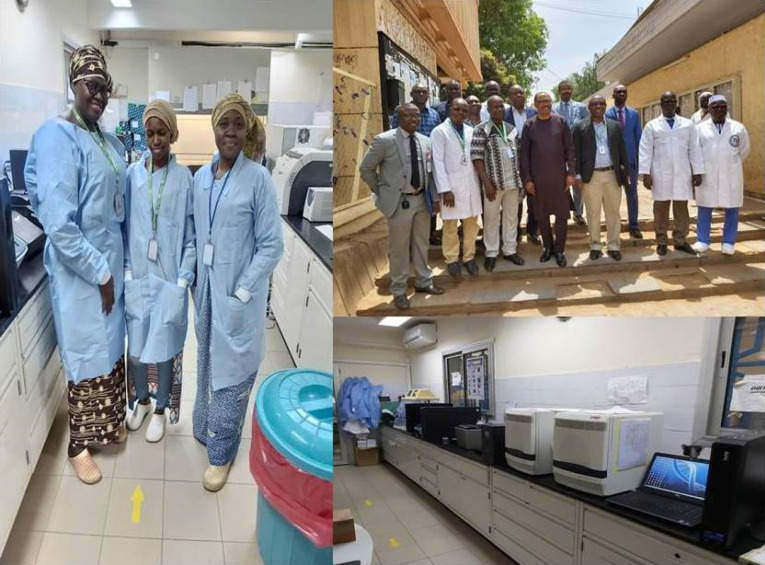
Well-equipped laboratories and wide range of collaboration and shared experience due to contagious infection have a critical role in control of COVID-19 infection in African countries. A wide network of laboratories and scientific communications participates important role in control of infection in these countries.

The laboratory diagnosis of COVID-19 in Mali benefited from an opportunity that allowed its anticipation, it is the expertise acquired in the context of the fight against the Ebola virus at the level of a university clinical research center (UCRC) of the Faculty of Medicine and Odontostomatology, which has a P3 level laboratory, as well as, an efficient molecular biology laboratory. The visit of an American researcher experienced in research on corona viruses, Professor Heinz Feldmann of Rocky Mountain virology laboratory of the National Institute of Health (NIH) the USA in early February 2020 allowed the transfer of competence for the realization of the RT-PCR of SARS-CoV-2 at this laboratory. The Ministry of Health has strengthened the capacity of the health system for diagnostic in its response plan of March 5, 2020, by identifying and strengthening the capacities of four laboratories for the RT-PCR diagnosis of COVID-19 with the support of partners, including the CDC in Atlanta. Therefore, the tests are carried out in four laboratories, namely those of the National Institute of Public Health (INSP), the UCRC, the Laboratory of Applied Molecular Biology (LBMA), and the Charles Merrieux Infectious Disease Center (CICM). The results are centralized by the INSP (Figure 2).

Niger, like other countries, has developed a national response plan against the spread of COVID-19 and a document of National Case Management Guidelines. Niger, through its national response plan envisaged a multisectoral response strategy. They do not know the date of the laboratory diagnosis.

On the strength of the experience acquired during the Ebola virus epidemic of 2014-2015, Guinea already had P3 type and molecular biology laboratories in place to deal with this new pandemic, COVID-19. The health authorities have reactivated the existing laboratories by strengthening the capacity of the health system for diagnosis in its response plan at the first warning signs by identifying and strengthening the capacities of the five laboratories. With the involvement of our partners, the existence of quality human resources and the availability of most laboratory equipment for the diagnosis by RT-PCR of COVID-19, the first cases were notified on site with the expertise local. Thus, all diagnostic tests are carried out in the five laboratories, namely: the INSP, the laboratory of the Pasteur Institute in Guinea, the Nongo hemorrhagic fever laboratory, the Epidemiology Research Center -microbiology and medical care (CREMS) of Kindia and the laboratory of the Center for Research and Training in Infectious Diseases of Guinea (CERFIG). The results of all these laboratories are centralized by the INSP.

## The clinical policy implemented for infection control

The coordination of activities to combat COVID-19 is ensured by the INSP. Mali has opted for the establishment of isolation centers in each health structure and at the entry points (borders, airports), where all suspect cases are isolated on site until the results of the COVID 19 test are returned. The positive cases are transferred to the care centers: each region has one at the regional hospital and Bamako the capital has 8 the CHUs of Point G, Gabriel Touré, Kati, the Dermatological Hospital, the Mali Hospital and two private polyclinics (Pasteur and Golden Life). These centers are structured with respect to the red, yellow, and green zones and are provided with material and equipment for the protection of patients and nursing staff. Human resources have also been mobilized and trained in order to apply the national guidelines for treatment that have been drawn up by the various learned societies (infectious diseases, resuscitators, pulmonologists, internists, gynecologists, pediatricians, etc.) and asymptomatic patients are treated in units for simple cases and severe and critical patients in intensive care units. Contact cases are sought and followed in self-containment by the health districts. However, The Nigerien subcommittee set up a follow-up file for hospitalized patients. The laboratory diagnosis comes to us on average 4 days after the sample. We don’t have a quick test. We apply PCRs carried out in the capital located 600 Kkm. For a month and a half, we have been able to perform PCR tests at our level. We do not have the ability to perform CT scans on our patients.

The coordination of activities to combat COVID-19 is ensured by the Ministry of Health and Public Hygiene through the ANSS. Guinean policy has been to set up epidemiological treatment centers either in health facilities or not and suspected or confirmed patients are isolated, either awaiting the result of the SARS-CoV-2 test or as part of therapeutic monitoring. For asymptomatic travelers arriving through Conakry Gbessia International Airport, a hotel complex is dedicated to monitoring during the 14 days awaiting their results. Symptomatic travelers are referred to the centre de traitement de l’épidémie (CT-EPI) COVID-19 for therapeutic management and diagnostic confirmation. As soon as, the COVID-19 test is positive by RT-PCR, all symptomatic or non-symptomatic patients are systematically hospitalized in one of the centre de traitement-

procédure pour les hôpitaux (CT-PPE). Each of this CT-PPE has a structure respecting the red, yellow, and green zones and equipped with materials and protective equipment for care providers and patients. All patients are referred to Donka National Hospital, where sorting will take place. Thus, severe or critical patients and symptomatic patients with or without comorbidities are directly admitted to intensive care and to the first floor, respectively. Most asymptomatic patients without comorbidities are confined to an extra-hospital CT-PPE for the Conakry area. For administrative regions, all centre de traitement de l’épidémie (CT-EPI) are intra-hospital. Human resources have also been mobilized and trained in order to apply the national guidelines for care, which have been drafted and validated by the various learned societies (infectious diseases, resuscitators, pulmonologists, internists, gynecologists, pediatricians, hygienists, etc.) and the partners, in this case, the WHO. Contact cases are sought and followed in self-containment by the competent health districts.

## Hospital Support and Diagnostic Tools

A follow-up file for hospitalized patients was initially developed by each structure and subsequently standardized with the support of WHO. This file is computerized with the District Health Information Software 2 (DHIS2, Health Information Systems Program at the University of Oslo) used by the national health information system (SNIS). The staffs have been trained to enter data on tablets. The pharyngeal samples are taken in the health districts and treatment structures (for follow-up tests) and are sent in triple packaging to the four designated diagnostic laboratories. The results took an average of 48 hours at the start currently the turnaround time for results is 24 hours. Chest CT has not been officially accepted for diagnosis, but in some facilities that have it and where hygienic conditions allow it, some patients have benefited from it. The chest X-ray was available at the intra-hospital CT-EPI in Conakry and performed at the request of healthcare providers. Other laboratory tests for inpatient follow-up were performed free of charge at the INSP laboratory, some of which were on a case-by-case basis.

The electrocardiogram was performed systematically in all patients with cardiovascular risks before they were started on hydroxychloroquine. Biological examinations (hematology, biochemistry, bacteriology, etc.) for monitoring hospitalized patients were carried out free of charge for patients with severe symptoms or with comorbidities at the private laboratory of BIOMAR 24 (BioMar Co, London, United Kingdom).

## Therapeutic Strategies

In all five countries, there are national guidelines documents. Several potential drugs were introduced during pandemic.^17^ It distinguishes simple cases from severe and critical cases, which are handled in different environments. The basic treatment is based on the use of chloroquine phosphate 200 mg every 8 hours for 10 days and azithromycin 500 mg per os in a single dose on the first day 250 mg/d from the second to the fourth day or ceftriaxone: 2g/d. For other comorbidities, specific treatments are used. Simple cases are those without: breathing difficulties; comorbidities (respiratory failure, chronic obstructive pulmonary disease, heart failure, asthma, renal failure, human immunodeficiency virus, viral hepatitis B and C, diabetes, etc); nor immunosuppressive treatment, corticosteroid therapy or anti-cancer. They prescribe paracetamol (1g x 3/d (as needed), vitamin C: 1g x 2/d (if asthenia), adapted rehydration, preventive anti-coagulation: systematic and double dose in patients in the inflammatory phase. Severe cases are represented by the presence of situations excluded in simple cases and the following objective criteria: respiratory rate >30/min; SpO_2_ <92% in ambient air; systolic blood pressure <90 mm Hg; altered consciousness; dehydration. For these serious cases in intensive care, the treatment consists of the administration of lopinavir + ritonavir (Kaletra): 200/50 mg x 2/day for 10 days associated with oxygenation with an extractor or non-invasive ventilation, heparin therapy, insulin therapy, corticosteroid therapy, antibiotic therapy and others as appropriate.

Criteria for recovery and discharge from conventional hospitalization is after 3 to 7 days without symptoms (apyrexia, FR <20 and no need for oxygen therapy for at least 48 hours). A first control PCR is carried out on Day 7 of hospitalization, if it is negative, a second PCR is carried out 48 hours later for confirmation.

## The lessons from this pandemic

- The population still had vivid memories of the barrier-hand washing gestures. Therefore, this facilitated the reactivation of already existing reflexes with strict respect for the measures enacted, then a gradual relaxation.
- The health structures, the sorting system initiated at their front door during the Ebola virus epidemic have made it possible to detect a number of cases preventing hospital transmission.
- The laboratory system, the teams trained and the equipment acquired during this same epidemic made it possible to make the diagnosis of SARS-CoV-2 very early in these countries.
- The test, trace, and treat strategy is effective before the community transmission phase. Absolute respect for barrier gestures remains a major element during the community phase.
- A sufficient human resource in number and quality, sufficient diagnostic and therapeutic management tools, and sufficient bedding capacity will allow a good response to an epidemic.

## Conclusion

In African countries, especially in these countries (Mali, Mauritania, Niger, Guinea, and Djibouti), due to the low international flights and low domestic travel, the spread and prevalence of COVID-19 was low and the return of the immigrants of these countries has caused the spread of COVID-19 among these countries. As well as, low communication and relationship culture among the people, disseminate the people in wide areas could be the causes of the low spread of COVID-19 in these countries. However, the roles of social distancing and social media are crucial and important in limiting the virus dissemination and fighting the pandemic with coordinate efforts with all civil society actors. Experience, preparation, and impact of previous infections epidemic such as the Ebola virus epidemic would have beneficial in these countries, which have promoted certain reflexes among people.

## Acknowledgments

This study was supported by several communities from African health organization. We thank all African participants during covid-19 pandemics and appreciate all efforts and successes. We hope to defeat COVID-19 in near future.

## Funding

This study was supported by several communities from African

health organization.

## Competing interests

None to declare.

## Ethical approval

This study was a commentary due to personal experiences by authors and no experience was done. But Helsinki declaration was considered for all steps of manuscript preparation. The authors claim that no part of this paper is copied from other sources.

## Authors’ contributions

All authors had the same participation in data collection and manuscript preparation and final approval of this study.
